# Changes in Anthropometric Measurements and Physical Fitness of Polish Students in 20-Year Period

**DOI:** 10.3390/ijerph192416885

**Published:** 2022-12-15

**Authors:** Jarosław Fugiel, Katarzyna Kochan-Jacheć, Dawid Koźlenia, Jarosław Domaradzki

**Affiliations:** Unit of Biostructure, Faculty of Physical Education and Sport, Wroclaw University of Health and Sport Sciences, Al. I.J. Paderewskiego 35, 51-612 Wroclaw, Poland

**Keywords:** secular trend, somatic traits, motor performance, adults

## Abstract

Background: Intergenerational changes are still being recorded worldwide, although their magnitude and direction may vary in different countries. The aim of this study was to determine changes in the magnitude and direction of changes in the body morphology and motor abilities of physically active adults over 20 years. Methods: Two hundred and fifty-two individuals aged 18–28 years volunteered to participate in the study. The changes were analyzed over a 20-year period (March 2001; P_1_ vs. March 2022; P_2_). The measured parameters were body height, weight, and body fat. Further, hand grip strength with dynamometer measurement, a sit-up test, a sit and reach test, and a standing long jump were performed to examine motor abilities. The results of the conducted tests were compared between subjects from both periods. Results: Our study confirms changes in trends concerning body morphology and motor ability performance. Higher values of body mass index and body fat were observed in P_2_ women, whereas these differences were not significant in men. Additionally, in terms of physical performance, the P2 group noted worse results than their peers from the past. Conclusion: Despite young adults claiming to have similar physical activity levels to those observed in the past, they demonstrate poorer physical performance and higher body fat levels. The observed changes can be considered negative.

## 1. Introduction

The assessment of intergenerational changes in body size and functional parameters in relation to secular trends in basic somatic features, body composition, and motor performance remains an important research problem. Observed intergenerational changes make it possible to create health policies and prepare preventive programs for different populations [1,2]. These changes are noted all over the world, although their dynamics or direction may vary in individual countries [3,4,5,6]. They depend on socio-economic and cultural changes and lifestyle, especially nutrition and physical activity [7,8,9].

Secular trends in many European countries and around the world continue to be observed. They will undoubtedly be studied in the upcoming decades and used to develop and update population norms for body size in populations. In most European countries, including Poland, body height has increased by 1 to 3 cm per decade over the last 150 years [10,11,12]. In Poland, secular trends in the adult population were most often analyzed based on the results of surveys of military recruits and university students. These measurements have been repeated since the 1950s [11,13]. The secular trend also indicates a dynamic increase in body weight, which has led to a rise in the BMI value [14,15]. This situation has increased the percentage of overweight and obese people in societies [16,17].

In the case of motor skills, a decrease in the level of its individual components has been observed in recent decades. However, studies of adults in relation to intergenerational changes are not so frequent [18,19,20]. Reducing the level of physical fitness results from a lifestyle change. Studies indicate a decrease in the level of physical activity and an increase in sedentary behavior [21,22]. The presented data also suggest that the Polish population is one of the least active societies in Europe [23,24]. This situation is worrying, and this low level of physical activity results in a reduced level of physical fitness and an increase in the number of overweight and obese people [25,26,27,28]. Hypokinesia is a serious problem in present times. It may lead to an increased risk of many diseases in civilization and a decrease in the biological condition of the population and future generations [29,30]. It should be noted that reduced body weight, as reflected in a low body mass index (BMI), may also have a negative impact on physical fitness, although research on the relationship between being slim and physical fitness is relatively limited [31,32]. Several studies have considered the relationship between physical fitness across the BMI spectrum in children and adolescents. The results generally indicate a curvilinear relationship; children and adolescents with a normal BMI tend to perform better in several elements of physical fitness compared to those with a low or high BMI [27,33,34,35].

## 2. Materials and Methods

### 2.1. Participants

The research was conducted in March 2001 (period 1; P_1_) and March 2022 (period 2; P_2_) by the Department of Biostructure, Wroclaw University of Health and Sport. The participants were recruited from students within the Faculty of Physical Education and Sport. The participants self-reported the amount of time they spent engaging in physical activities in their leisure time and practicing sport. The exclusion criteria in both examinations were: activity in sport, sick leave in the two weeks before examinations, and any injuries in the six weeks before the assessments (17 students were excluded from the studies). Finally, two hundred and fifty-two individuals aged 18–28 years volunteered to participate in the study. In P_1_, 70 men (M) and 78 women (W) were assessed, whilst in P_2_, 55 men (M) and 49 women (W) were assessed.

### 2.2. Procedures

The research was carried out in the Biokinetics Research Laboratory of the Wroclaw University of Health and Sport Sciences, which has a Quality Management System Certificate (PN-EN ISO 9001: 2009; Certificate Reg. No. PW-48606-10E). All the participants were assessed by staff members of the Department of Biostructure in the Wroclaw University of Health and Sport Sciences. The assessments were performed in the morning, and all the participants were asked to refrain from eating, drinking, and exercise for at least 3 h prior to the assessments.

### 2.3. Anthropometry

Height and weight were measured using the procedures of Martin and Saller [25]. A Swiss anthropometer (GPM Anthropological Instruments, DKSH Ltd., Zürich, Switzerland) was used for height measurements (BH). A SECA model M799 device (approval type D07-09-032, Hamburg, Germany) was used to measure body weight (BW). The measurement accuracy was 0.1 kg for mass and 0.1 cm for height.

Triceps and abdominal skinfolds were measured to the nearest 0.1 mm using a Harpenden Skinfold Caliper. The two skinfolds were used to calculate the body fat percentage (BFP). The Harpenden Skinfold Caliper and the reproducibility of subcutaneous fat measurement were positively examined [36]. The equations of Slaughter et al. [37] were used to predict the BFP for the comparative analysis.

Participants’ anthropometric measurements were taken individually in a private room. The examiners were experienced researchers with several years of experience (at least 5 years of experience and participation in research) and performed the measurements in both 2001 and 2022.

### 2.4. Musculoskeletal Fitness

Before the motor performance tests, the students were asked to change into sports clothes. Four fitness tests were administered: 1. hand strength (HS)—hand grip strength was measured with an accuracy of 1 kg by means of a JAMAR hydraulic hand dynamometer (Sammons Preston Rolyan, Bolingbrook, IL, USA) with the adjustable handle set to position two (the best of two trials was used for the analysis); 2. abdomen strength (ABS)—the number of complete sit-ups performed in 30 s was recorded (one trial was performed); 3. flexibility (FL)—a sit and reach test was performed: with their palms facing downwards and their hands on top of each other, the participant reached forward as far as possible along the measuring line (one trial was performed); 4. leg power (SJ)—a standing long jump was performed, measured as the distance from the front edge of the takeoff line to the heel nearest to the takeoff line following a maximal jumping effort (the best of two trials was used for the analysis).

### 2.5. Statistical Analysis

The numbers of participants who engaged in physical activity in their leisure time (in addition to physical activity during mandatory lessons in University) are presented as percentages. Pearson’s chi-squared test (*χ*^2^) was used to determine whether there was a statistically significant difference between the expected frequencies and the observed frequencies in the PA and NA groups during P_1_ and P_2_, with men and women analyzed separately.

The Shapiro–Wilk test was used to evaluate the normality of the continuous variables’ distribution. Descriptive statistics for continuous measurements (age, BH, BW, BMI, BFP, HS, ABS, SJ, and FL) are presented as mean and SD with a 95% CI and were calculated separately for men and women for each period (P_1_ and P_2_).

A two-factor analysis of covariance (ANCOVA) was used to evaluate differences between groups (period—groups examined in 2001 and 2022; and sex—men and women). Continuous measurements were used as dependent variables (DVs). Age was used as a confounding variable (CV). When significant differences were observed (significant F-ratio), a post hoc test (Tukey’s HSD) was used to determine pairwise differences.

The relationships between the results for motor tests (HS, ABS, SJ, and FL) and BFP were determined using simple regression analysis using y = b_0_ + b_1_ × BFP, where b_0_ was the constant (intercept) and b_1_ was the regression coefficient (slope). The results are presented in graphical form as scatterplots. Regression equations are included in the figures.

Two regression lines significantly shift up or down if the constants (intercepts) of two regressions are significantly different, thus confirming different levels of the relationship between the variables. To test the difference between constants, multivariate regression analysis (MRA) was used. The angle of the regression line depends on the regression coefficient (slope). When the slope coefficients of two regression lines are different, a one-unit change in a predictor is associated with different mean changes in the response. This means a different intensity of the relationships is presented by the two lines. To test the slopes, the same approach as mentioned above was used with an interaction term included in the model. The coefficients and corresponding p-values for the tested intercepts and slopes are presented in the tables.

Statistical significance was set at α = 0.05. We used Statistica version 13.0 (StatSoft Polska, Cracow, Poland 2022) for data analysis.

## 3. Results

Initially, the chi-squared test was performed to test equality in proportions between active and non-active student across periods. The results indicated a lack of significant differences in the number of physically active (PA) and not physically active (NA) participants during both periods and for both sexes (Table 1). Out of 70 men in P_1_ and 49 in P_2_, about 80% were PA. Out of 78 women in P_1_, 50 (64.1%) were PA, whilst out of 55 women in P_2_, 33 (60.00%) were PA. These associations were not statistically significant (men: *χ*^2^ = 0.049, *p* = 0.824; women: *χ*^2^ = 0.231, *p* = 0.630).

The descriptive statistics of the measured parameters (anthropometrical measurements and motor tests) are presented in Table 2.

The results of ANCOVA, presented in Table 3, suggested secular changes in all the measurements, except BH (F = 0.100, *p* = 0.738), whereas sex was a factor that statistically significantly influenced all the parameters. Detailed comparisons using post hoc tests are presented in Table 4.

The post hoc tests confirmed a lack of changes in BH in men and women. Intergenerational differences in BW, BMI, and BFP were observed in women but not in men (Table 4).

Generally, the participants examined in P_1_ (men and women) demonstrated greater power and flexibility. The mean values for strength (HS and ABS), SJ, and FL were greater compared to the participants assessed in P_2_. However, not all differences were statistically significant (Table 4). The men studied in P_1_ were stronger (HS and ABS) and had greater flexibility, but there were no significant differences in SJ. Meanwhile, the women examined in P_1_ achieved significantly better results in ABS and SJ (Table 4).

**Figure 1:**
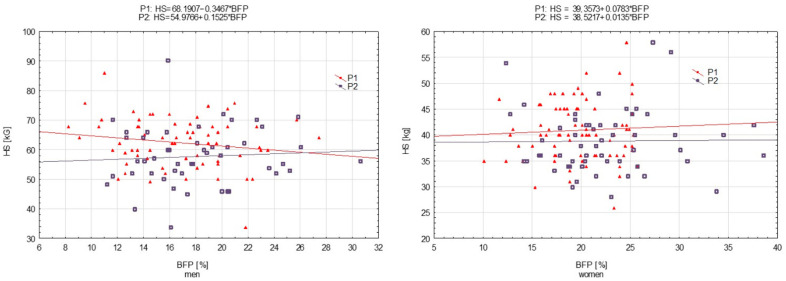
Hand strength (HS) measured in 2001 (P_1_) and 2022 (P_2_) regressed on body fat percentage (BFP)—men and women, respectively.

The relationship in the case of HS was greater in the men’s group assessed during P_1_, whilst in the rest of the groups, associations were weak (Figure 1), as confirmed by the horizontal lines. The constants of the regression functions (included in the scatterplots) were different (in men and women), which confirmed statistically significant coefficients calculated from multivariate analysis (Table 5). However, statistically not significant interactions suggested that one-unit changes in predictors were associated with similar mean changes in response, as in P_1_ and P_2_ for both men and women.

**Figure 2:**
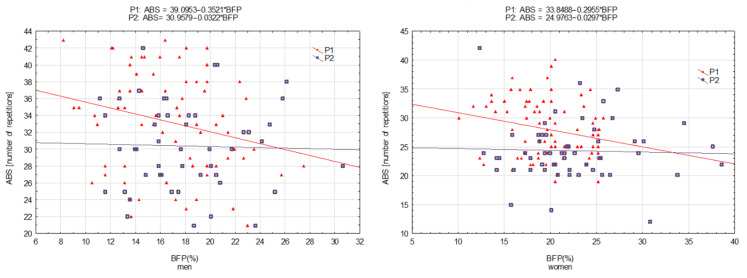
Abdomen strength (ABS) measured in 2001 (P_1_) and 2022 (P_2_) regressed on body fat percentage (BFP)—men and women, respectively.

**Figure 3:**
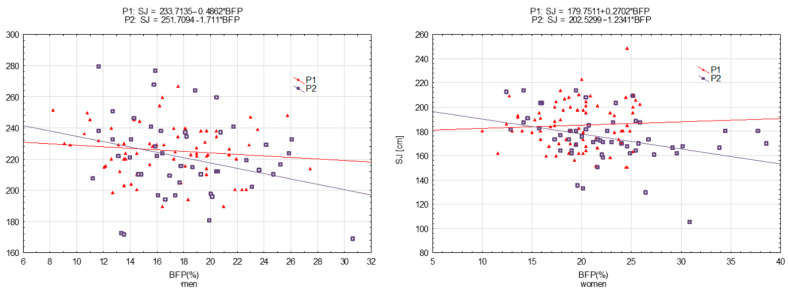
Power of legs (SJ) measured in 2001 (P_1_) and 2022 (P_2_) regressed on body fat percentage (BFP)—men and women, respectively.

**Figure 4:**
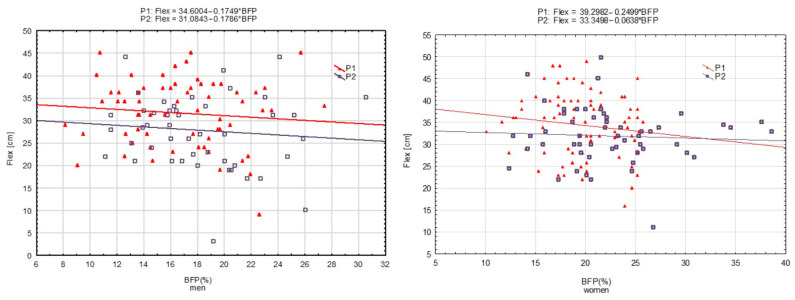
Flexibility (Flex) measured in 2001 (P_1_) and 2022 (P_2_) regressed on body fat percentage (BFP)—men and women, respectively.

Figure 1, Figure 2, Figure 3 and Figure 4 present motor performance tests regressed on BFP.

Similarly, statistically significant shifts (for men and women) were observed in ABS. In the case of this test, the relationship with BFP was greater in P_2_ (Figure 2). However, the slope coefficients were not different (Table 5).

There was a significant shift in SJ, but only in women (Figure 3 and Table 5). In addition, there were significant differences in slopes. In the scatterplot, it appears that a one-unit increase in BFP was associated with a significantly greater decrease in SJ during P_1_ compared to during P_2_ (Table 5).

In the case of FL, a significant shift was only observed in women. The coefficients calculated for the assessment of the differences between slopes were not statistically significant in either men or women (Table 5).

## 4. Discussion

Our study has revealed changes in morphological and functional parameters in young adults over 20 years and the impact of BFP on functional abilities. Comparable physical activity levels in groups from both periods were observed. For BH, there was no trend. Sex strongly affected the observed differences in body weight, BMI, and BFP, with higher values in women, whereas in men, these differences were not significant. Generally, the men and women in P_2_ performed worse in the physical performance tests. A higher BFP negatively impacted motor abilities, especially in the P_1_ group, but in P_2_, lower fitness levels were not strongly associated with BFP.

The decrease in physical activity levels observed in the various age groups and hometown populations is associated with an increased risk of developing many diseases [38]. Moreover, this process has been accelerated due to the COVID-19 pandemic [39]. This decrease in physical activity levels is also associated with reduced physical fitness [38]. On the other hand, overall well-being, access to food, and technological developments are related to morphological development expressed by increased body height and weight, which is also associated with more body fat. The problem concerning increased body weight and body fat among young adults was visible several years ago, and it is still present [12]. Kalka et al. [12] observed a 3 kg gain in body weight and a 3.1% increase in the percentage of body fat during 1996–2012 in Polish male students. These results met our study results, where the change in men’s body weight was 2 kg, and the change in the percentage of body fat was 2%. It has been shown that increased body fat is associated with higher cardiovascular disease risk [40]. Our results showed that currently, men and women have a higher BMI and BFP than their peers in the past, which can be seen as negative. Similar to us, Robic-Pikel [41] observed increased body fat over 70 years in young adults in Slovenia. In another European population, Lundbland et al. [42] also observed negative trends in body composition in older adults. This suggests that younger subjects may continue to gain fat throughout their life, worsening their health status and life quality [43]. Additionally, obesity is visible outside Europe and is a global problem [44]. The solution seems to be physical activity, but negative trends in this area have been observed.

Our results show comparable physical activity levels; so, it is possible that decreased physical fitness is associated with lifestyles that incorporate less locomotion or physical work compared to those in the past. Our study found similar results to Pribis et al. [18]. They showed negative changes concerning decreased physical fitness and an increase in body fat in college students.

In Poland, Podstawski and Zurek [45] studied male university students between 2000 and 2018 in two-year intervals. Generally, the trends the authors found are convergent with our results. There was trend of increased body weight and BMI over two decades. In both studies, body mass changed by 2–3 kilograms (our studies: body mass—77.32 in 2001 and 79.14 in 2022; students from Warmia and Mazury—76.38 in 2000 and 79.72 in 2018). Changes in body height (1.2 cm in our study; 1.7 cm in Podstawski and Zurek’s study) resulted in lower changes in body mass index than in body mass (our study: BMI—24.00 in 2001 and 24.28 in 2022; Podstawski and Zurek’s study: BMI—23.50 in 2000 and 24.07 in 2018). In addition, our results are convergent in relation to motor performance. They pointed out that participants studied in 2018 achieved poorer results in a motor test. This includes the strength of the abdomen muscles (our study: 33 sit-ups in 2001 and 30 in 2022; Podstawski and Zurek: 26 sit-ups in 2000 and 24 in 2018), the power of the legs (our study: 226 cm in 2001 and 220 cm in 2022; Podstawski and Zurek: 215 cm in 2000 and 211 cm in 2018), and flexibility (decrease of 4 cm in the flexibility test in our studies; 1.5 cm decrease in Podstawski and Zurek’s).

An increase in body composition indices and lower physical fitness in college students in the USA was shown by Wetter et al. [46]. Some authors suggest that the negative impact of body fat on physical fitness is linked to decreased cardiorespiratory fitness [47]. It often has further consequences related to metabolic disorders and leads to worsening health [37]. Appropriate physical activity and nutrition could stop these negative changes. In some special populations, positive changes have been observed. A study by Knapik et al. [20] summarized the data from almost 40 years based on a military population and showed an increase in strength over 40 years.

We are aware that our study has some limitations. The first is procedure of the assessment of the body fat percentage of students with Slaughter regression equations [37]. The results of many studies demonstrated that these equations were accurate for non-obese children and adolescents. Therefore, the body fatness of the study sample could have been overestimated [48]. The next limitation is the interpretation of the relationship between BMI and body fat percentage. A known problem related to BMI is that the assessment of body fat content based on body height and weight is inaccurate. We do not have data in ten-year intervals (there was a lack of data from the years 2010–2011), which would have added further insight into the observed trends. Additionally, our findings only relate to physical education students. On the other hand, the study also has some strengths. We presented unique data in this scientific area. We compared the functional and morphological aspects of a similar population and provided comprehensive observations.

## 5. Conclusions

Our study found changes concerning body morphology and motor abilities. However, the observed changes can be considered negative. Despite young adults claiming to have similar physical activity levels as those observed in the past, they now achieve worse results in physical performance tests and have more body fat. It is possible that the development of technology supports these negative trends. Therefore, we need to counter the progressive loss of physical fitness and, due to its direct association with cardiovascular disease, decrease body fat. There is a need to educate people regarding nutrition and encourage physical activity.

## Figures and Tables

**Table 1:** Numbers (percentages) of the physically active and non-active students. Pearson’s *χ*^2^ test results.

Group	Men		Women	
Period	P_1_	P_2_	P_1_	P_2_
Physically active	56 (80.00%)	40 (81.63%)	50 (64.10%)	33 (60.00%)
Not physically active	14 (20.00%)	9 (18.37%)	28 (35.90%)	22 (40.00%)
*χ*^2^ statistics	*χ*^2^ = 0.049, *p* = 0.824		*χ*^2^ = 0.231, *p* = 0.630	

**Table 2:** Descriptive statistics of the measured parameters in periods and sex categories.

Variable	Men P_1_				Men P_2_			
	x	−95%	95%	SD	x	−95%	95%	SD
Age	23.54	23.24	23.84	1.24	20.66	20.14	21.18	1.81
BH	179.22	177.45	180.99	7.42	180.49	178.50	182.48	6.94
BW	77.32	74.89	79.74	10.18	79.14	76.29	82.00	9.93
BMI	24.00	23.50	24.49	2.08	24.28	23.55	25.00	2.53
BFP	16.75	15.76	17.73	4.13	18.35	17.00	19.70	4.71
HS	62.39	60.40	64.37	8.33	57.78	55.01	60.54	9.63
ABS	33.20	31.76	34.64	6.03	30.37	28.88	31.85	5.16
SJ	225.57	221.63	229.51	16.52	220.31	212.81	227.80	26.10
FL	31.67	29.88	33.46	7.51	27.81	25.42	30.19	8.31
**Variable**	**Women P_1_**				**Women P_2_**			
Age	23.47	23.17	23.76	1.30	20.02	19.80	20.24	0.80
BH	168.32	167.00	169.65	5.87	167.65	165.59	169.70	7.60
BW	58.90	57.27	60.52	7.19	63.53	61.13	65.92	8.86
BMI	20.75	20.32	21.17	1.88	22.59	21.84	23.34	2.78
BFP	19.66	18.80	20.52	3.80	22.44	20.87	24.01	5.81
HS	40.90	39.60	42.19	5.75	38.82	37.15	40.50	6.20
ABS	28.04	26.91	29.16	4.97	24.31	22.93	25.69	5.11
SJ	185.06	180.89	189.24	18.50	174.84	169.24	180.44	20.71
FL	34.38	32.75	36.02	7.25	31.92	30.21	33.63	6.32

Footnote: BH—body height, BW—body weight, BMI—body mass index, BFP—body fat percentage, HS—hand strength, ABS—abdomen strength, SJ—standing jump, FL—flexibility.

**Table 3:** Results of ANCOVA (F-ratio and corresponding *p*-values).

Measurement	ANCOVA	Period Effect	Sex Effect
Body height	F	0.100	178.900
	p	0.738	**0.000**
Body weight	F	7.820	217.410
	p	**0.006**	**0.000**
Body mass index	F	13.130	71.070
	p	**0.000**	**0.000**
Body fat percentage	F	14.046	35.770
	p	**0.000**	**0.000**
Hand strength	F	12.160	445.170
	p	**0.001**	**0.000**
Abdomen strength	F	22.930	66.657
	p	**0.000**	**0.000**
Standing jump	F	8.970	276.110
	p	**0.003**	**0.000**
Flexibility	F	11.287	13.115
	p	**0.001**	**0.000**

Statistically significant (*p* < 0.05) in bold.

**Table 4:** Post hoc test results (*p*-values).

Variable	P_1_–P_2_		Men–Women	
	Men (2–4)	Women (1–3)	P_1_ (1–2)	P_2_ (3–4)
Body height	0.760	0.946	**0.000**	**0.000**
Body weight	0.697	0.018	**0.000**	**0.000**
Body mass index	0.915	0.000	**0.000**	**0.001**
Body fat percentage	0.233	0.003	**0.001**	**0.000**
Hand strength	**0.005**	0.393	**0.000**	**0.000**
Abdomen strength	**0.023**	**0.000**	**0.000**	**0.000**
Standing jump	0.499	**0.021**	**0.000**	**0.000**
Flexibility	**0.025**	0.226	0.112	**0.023**

Statistically significant (*p* < 0.05) in bold.

**Table 5:** Statistics (b-coefficients and corresponding *p*-values) derived from multivariate regression analysis to test statistical significance of the differences between intercepts and slopes calculated in simple regressions for relationships between motor tests and BFP in men and women examined in P_1_ and P_2_.

Parameter	Motor Test	Men		Women	
		b	p	b	p
INTERCEPT (constant)	Body height	−4.43	0.010	−2.18	**0.048**
	Body weight	−2.51	0.021	−3.37	**0.000**
	Body mass index	−3.55	0.363	−8.38	**0.019**
	Body fat percentage	−3.58	0.017	−2.09	0.100
SLOPE (coefficient)	Hand strength	0.50	0.187	−0.06	0.776
	Abdomen strength	0.32	0.182	0.32	0.182
	Standing jump	−1.22	0.160	−1.50	**0.040**
	Flexibility	0.00	0.991	0.19	0.479

Statistically significant (*p* < 0.05) in bold.

## Data Availability

The data presented in this study are available on request from the corresponding author.
